# Integrating Surveillance and Climate Data for Cholera Early Warning in Ethiopia

**DOI:** 10.5334/aogh.4742

**Published:** 2025-09-13

**Authors:** Hailemichael B. Dadi, Desalegn T. Negash, Sisay W. Adall

**Affiliations:** 1 Saint Paul’s Hospital Millennium Medical College, School of Public Health, Addis Ababa, Ethiopia; 2 Ethiopian Meteorological Institute, Health Meteorology and Insurance Index, Addis Ababa, Ethiopia; 3 Ethiopian Public Health Institute and Resolve to Save Lives, Addis Ababa, Ethiopia

**Keywords:** cholera, climate change, sentinel surveillance, disease outbreaks

## Abstract

*Background:* Ethiopia faces persistent cholera outbreaks worsened by increasing droughts and heavy rainfall due to climate change. More than 15.9 million Ethiopians reside in districts historically prone to severe cholera outbreaks. There have been efforts to enhance cholera surveillance by integrating it with climate data and prioritizing forecasting to improve adaptation.

*Objectives:* This study aimed to investigate climate adaptation measures, explore temporal associations between climate variables and cholera incidence across Ethiopian districts, and identify observed thresholds and potential climate indicators for enhancing early warning systems.

*Methods:* We conducted a literature review and secondary analysis of climate-cholera data. Temporal patterns and lagged effects of temperature and rainfall on cholera were examined using descriptive statistics, Pearson correlation, and time-lag analysis (up to three weeks). To determine optimal outbreak conditions, we assessed historical temperature and rainfall averages to measure anomalies. Data visualization, including line graphs, time series plots, and heatmaps, was performed using MS Excel and R.

*Findings:* District-specific temperature and rainfall variations and thresholds were identified. The analysis dataset included 2,298 cholera cases across 13 districts. Cholera transmission exhibited distinct patterns: a monomodal pattern in five districts with primary peaks during the wet season (June–September), driven by heavy rainfall, and a bimodal pattern in eight districts with secondary peaks during the secondary wet season (February–May). Most outbreaks occurred between epidemiological weeks 10 and 42, with 63.7% of cases in weeks 29–42. Rainfall strongly correlated with cholera in monomodal districts, while temperature showed broader correlations in bimodal districts.

*Conclusions:* Understanding district-specific variations in temperature and rainfall is crucial for managing cholera outbreak risks. These insights can inform early warning systems by providing essential indicators for potential outbreaks. Strengthening epidemiological forecasting capabilities, particularly in drought- and flood-prone regions, can support the cholera early warning system, enabling more timely and proactive interventions.

## Background

Ethiopia is the second most populous country in Africa, with an estimated 126.5 million people in 2023 [1]. Nearly 80% of the population lives in rural areas, primarily relying on subsistence agriculture [2]. The country experiences three meteorological seasons based on rainfall distribution. Kiremt (June–September) is the wet season, accounting for 50–80% of annual rainfall. Bega (October–January) is the dry and windy season with less rainfall. Belg (February–May) is the second rainy season with occasional showers [3]. Annual rainfall distribution ranges from approximately 2,000 millimeters (mm) in the highlands to less than 300 mm in the lowlands, with a mean of 815.8 mm [4]. The country’s mean annual temperature is 22.6°C [5]. Access to water, sanitation, and hygiene (WaSH) remains a significant challenge in Ethiopia. Currently, 31% of the population and 40% of rural households still access water from unimproved sources [2], 80% use unimproved toilet facilities, and 27% have no facilities at all [6]. Cholera is influenced by a complex interplay of factors beyond WaSH. Social, economic, and behavioral factors, institutional factors, and climate change factors all play key roles [7]. Cholera is endemic in Ethiopia, contributing to a substantial burden of diarrheal diseases. While the number of cholera cases has fluctuated over the years, 2023 saw the highest number of cases in the past five years. Currently, over 15.9 million Ethiopians reside in districts that have been repeatedly and extensively impacted by cholera outbreaks, subsequently putting them at high risk for future outbreaks as well [7]. Since 2019, 46,097 cholera cases and more than 600 deaths have been documented with a cumulative case fatality rate (CFR) of 1.6%, surpassing the standard 1% [8].

### Cholera and climate variability

Cholera is an acute diarrheal disease caused by the bacterium *Vibrio cholerae* (serotypes O139 or O1). Infection commonly occurs through the fecal-oral route, mainly from surface and shallow wells [9]. Climate determines the seasonal and inter-seasonal patterns of cholera outbreaks [10]. Cholera is significantly affected by the El Niño Southern Oscillation (ENSO) which significantly influences climate patterns that can affect cholera incidence [11, 12] by increasing ocean and sea surface temperatures (SST), leading to extreme weather events and anomalies [13]. Rising local temperatures correlate with inter-annual cholera variability, as higher ambient temperatures raise water temperatures, promoting pathogen growth [11]. Increased rainfall can cause flooding, altering salinity, pH, and nutrients, fostering algal blooms that aid bacterial proliferation. Studies have reported that excess waterborne diseases, including cholera risk, are associated with heavy rainfall events, particularly following dry periods and during flooding events [14–17]. Flooding runoff facilitates the mobilization of pathogens into surface water and unprotected groundwater sources, leading to higher incidences of cholera outbreaks during the rainy season as these sources become overwhelmed [5, 14, 15, 18]. In contrast, dry seasons or droughts create ideal environmental conditions for *V. cholerae* to multiply rapidly and limit access to safe water, leading to poor hygiene and sanitation [10, 11, 19]. While *V. cholerae* thrives in high temperatures, moderate levels are often optimal. Prolonged favorable conditions typically involve a lag, delaying the increase of bacterial levels, human exposure, symptom onset, and outbreak reporting [10, 11].

The Intergovernmental Panel on Climate Change (IPCC) sixth assessment reported that climate-induced increase in temperature, rainfall, and flooding has already led to a rise in cholera incidence [20]. Climate change poses significant health risks to Ethiopia’s population [5]. Studies in Ethiopia have shown a strong correlation between cholera outbreaks and meteorological parameters, including seasonality [14]. In Ethiopia, El Niño has been linked to drought during the main rainy season and increased rainfall in the short rainy season, while La Niña has been associated with higher-than-average rainfall in the main rainy season [21]. This climatic variability affects the dynamics, spatial distribution, and temporal range of *V. cholerae*, influencing its exposure routes [22], bacterial growth, and survival [15, 17]. Average temperatures in Ethiopia have increased by 1°C since 1960, at an average rate of 0.25°C per decade [5]. Compared to central and northern highland areas, the eastern and southeastern regions of Ethiopia are expected to experience the most significant temperature increases. Projection studies indicate that mean monthly temperatures are anticipated to increase by 1.8°C by the 2050s under a high-emission scenario [5] (Table 1). Additionally, the number of “hot” days (i.e., above 35°C) is expected to continue increasing [4]. Temperature-induced drought is the most significant and recurring climate hazard in Ethiopia, particularly for pastoral and agro-pastoral communities inhabiting drought-prone areas [5]. These events are expected to become more frequent [18]. This increased likelihood of aridity and drought stress has been linked to severe water scarcity in some regions [5], contributing to cholera outbreaks [18]. Unlike temperature, precipitation projection trends across Ethiopia exhibit high degrees of uncertainty, as well as inter-annual and spatial variability [5, 18, 23]. Projected trends indicate a potential increase in the southwest and southeast areas, while northern regions are generally anticipated to experience a decrease in rainfall [5]. According to World Bank projections, there is likely to be an increase in extreme high rainfall events through the end of the century [5] (Table 1).

**Table 1:** CMIP5 ensemble projection of annual temperature and precipitation, under RCP8.5 high emission scenario, Ethiopia.

CMIP5 ENSEMBLE PROJECTION	2020–2039	2040–2059	2060–2079	2080–2099
Annual temperature	**+0.6 to +1.5**	**+1.2 to +2.6**	**+2.1 to + 4.0**	**+2.8 to +5.5**
Anomaly (°C)	(+1.0 °C)	(+1.8 °C)	(+2.8 °C)	(+3.7 °C)
Annual precipitation	**-14.4 to +21.2**	**-16.8 to +27.4**	**-18.8 to 37.6**	**-17.5 to +50.0**
Anomaly (mm)	(+2.2 mm)	(+3.1 mm)	+6.0 mm	+9.7 mm

*Note: Bold value is the range (10*^th^ – *90*^th^ *percentile) and values in parentheses show the median.*

*Source: Adapted from Climate Risk Profile: Ethiopia (2021): The World Bank Group* [5].

Despite recognizing climate change as a key driver of cholera outbreaks, there is a significant knowledge gap regarding implementation of climate adaptation measures. In Ethiopia, limited research has focused on specific strategies to mitigate climate-related risks. Existing literature primarily emphasizes public health responses, such as outbreak investigations and vaccination campaigns, with little exploration of the connection between cholera trends and climate adaptation. Additionally, the role of cholera early warning systems (EWS) and forecasting, particularly with climate variables, remains largely unexamined. While studies link climate variability to cholera, district-specific patterns in Ethiopia remain underexplored, hindering targeted EWS development.

### Climate change adaptation strategies

**Figure 1:**
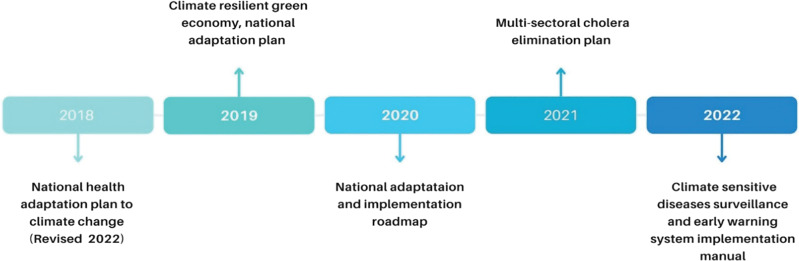
Timeline of key climate adaptation strategies for cholera in Ethiopia.

Impacts on human health and health systems from climate variability and change can be reduced or avoided through well-designed adaptation measures [24]. According to the IPCC, cholera risk is projected to rise if climate change continues along the representative concentration pathways scenarios without effective adaptation strategies [15]. Ethiopia is particularly vulnerable to climate variability due to its low adaptive capacity [5], prompting the country to initiate several adaptation strategies aligned with Sustainable Development Goal (SDG) 13.2, which focuses on integrating climate change measures into national policies [25]. A critical adaptation measure related to cholera is the improvement of EWS. The EWS within the Integrated Disease Surveillance and Response employs both event-based surveillance and indicator-based surveillance to monitor risks and prioritize diseases [7]. In 2014, Ethiopia developed key national policy frameworks to address the looming threat of climate change [26]. Subsequently, in 2015, the Ethiopian Ministry of Health and the World Health Organization Ethiopia conducted the first climate vulnerability and adaptation assessment of health in relation to climate change [27]. The assessment outlined key adaptation strategies targeted at Climate Sensitive Diseases (CSD), which included: (1) improving surveillance systems by enhancing sensitivity in detecting CSD; (2) integrating environment and health surveillance; risk mapping; and establishing EWS, particularly for CSD; (3) strengthening EWS by shifting the focus from response to greater emphasis on forecasting and prevention; (4) establishing a health and climate data management system to strengthen data use by integrating climate and health data [27]. In 2018, Ethiopia developed the National Health and Adaptation Plan for climate change, reflecting the strategies outlined by the prior assessment [18] (Figure 1).

Cholera is one of the 23 diseases prioritized by Ethiopia’s Public Health Emergency Management (PHEM) system [6, 7]. As part of the efforts to eliminate cholera, the Ethiopian National Cholera Elimination Plan (2021–2028) guided a sensitivity hotspot analysis that identified 118 districts (*woreda*) across 10 regions and city administrations as high-priority hotspots based on historical epidemiological data [7]. Given the strong correlation between weather variability and patterns of excess cholera risk, it is crucial to construct a climate-based EWS by integrating climate data with cholera incidence information [3, 14, 28]. In 2017, Ethiopia launched a CSD surveillance and early warning project, establishing 14 sentinel surveillance sites to monitor 5 prioritized diseases, including cholera at 10 sites [3]. These sites serve as designated health facilities for early reporting of priority events, providing high-quality case-based data that complements community and event-based surveillance [7]. The data are crucial for monitoring cholera risk factors along with reports from the local meteorological indicators regularly reported to the Ethiopian Public Health Institute (EPHI), informing the EWS and aiding in forecasting potential cholera outbreaks [7].

In Ethiopia, surveillance data for cholera, weather, and environmental factors are collected from various sources with different reporting frequencies. Weekly cholera data and cholera immunization coverage are reported by district CSD focal points. Weather-related data, such as total rainfall, relative humidity, maximum temperature, and minimum temperature, are also collected weekly by the Ethiopian Meteorological Institute (EMI) through its local branch stations. Environmental data, including district open defecation free, latrine coverage, and utilization rate, are reported yearly by the Ministry of Health. District safe water coverage is reported annually by the Ministry of Water and Energy. Population shape files are compiled by the Central Statistics Agency, and data on internally displaced people are reported by district CSD focal points [3].

While sentinel surveillance sites are crucial for monitoring cholera outbreaks, their effectiveness is significantly enhanced with active community involvement. This is because effective surveillance systems require continuous data collection and timely compilation. In this regard, Community-Based Surveillance enables communities located near the outbreaks to actively provide their input to the cholera surveillance by reporting outbreaks and rumors to sentinel health facilities. Such reporting facilitates immediate response actions and triggers investigations by rapid response teams [7]. Building on these adaptation efforts, this study aims to investigate climate adaptation measures, explore temporal associations between climate variables and cholera incidence across Ethiopian districts, and identify potential climate indicators for EWS enhancement.

## Approach

### Study design and analysis

This case study utilized document review and secondary data analysis. We conducted a comprehensive review of peer-reviewed and grey literature. Epidemiological cholera data were collected from the EPHI, the PHEM surveillance, and CSD databases. Weather projection data were accessed from the World Bank’s historical and climate projections. We also obtained additional meteorological data, including average temperature and total rainfall measurements from EMI. ENSO global data were gathered from the National Weather Service/CPC for the Niño 3.4 region (5°N-5°S, 120°-170°W), which governs the climate system. Data from EPHI and EMI sources were integrated and aligned on a weekly basis, while ENSO and clinical case data were integrated and aligned on a monthly basis to facilitate analysis.

We conducted a descriptive analysis to examine the temporal patterns and trends. Simple time series plots were used for cholera incidence, temperature, and rainfall. We cross-referenced key periods of interest, such as peaks in cholera cases, with corresponding temperature and rainfall data to investigate any temporal correlations. In addition, heatmap plots were generated to identify potential patterns and associations between cholera incidence and environmental factors on a temporal scale.

The Pearson correlation coefficient (r) was used to measure the strength and direction of the relationship between cholera incidence and weather variables. The data analysis and visualizations were conducted using MS Excel and R statistical software, leveraging libraries such as ggplot. We conducted a lag and correlation analysis to determine the time lag between temperature, rainfall, and cholera cases. This method was chosen as sufficient for exploratory analysis in a data-limited study setting where covariate data are scarce, though it cannot capture non-linear climate-cholera relationships. We examined lag times from Lag 0 to Lag 3 (up to three weeks) [11, 16]. This was guided by evidence from similar endemic cholera settings with poor WaSH coverage where short-term climate effects on transmission typically manifest within this timeframe. Identifying significant deviations from normal weather patterns is vital for understanding how unusual weather may influence cholera outbreaks. Thus, we performed an anomaly analysis using historical data on cholera cases and weather variables.

## Findings

### Sentinel sites and districts included

We used a predetermined criteria of the number of reported cholera cases in the past two years, and diverse geographical and weather variability representing diverse agro-ecological districts, arid pastoralist lowlands, highland and agrarian zones, and urban centers. Seven out of 10 regions and 13 of 38 districts with sentinel sites were purposively included as study areas. The sentinel sites which were included in the final analysis and the corresponding selected regions and districts are: Afar: Dubti and Semera Logia town; Amhara: Kombolcha town, Dessie Zuria, Bahir Dar Zuria, and Dera; Harari: Jinela and Hakim; Sidama: Hawassa and Wondo Genet; Somali: Gode; South Ethiopia: Dilla Zuria; and Dire Dawa town administration.

### Climate-Cholera associations

**Figure 2:**
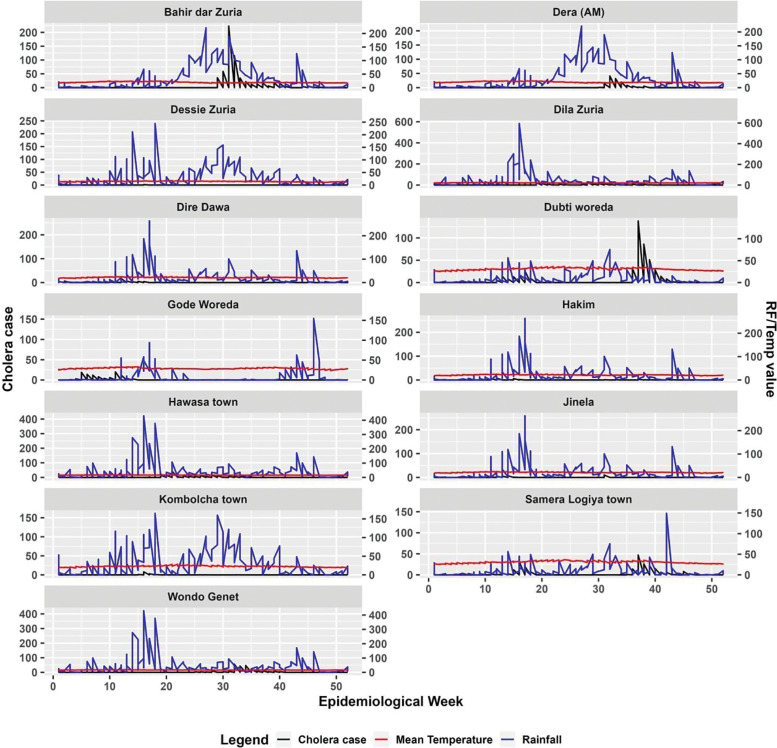
Cholera case distribution in relation to rainfall and temperature across study districts in Ethiopia.

We conducted a descriptive analysis to investigate the possible association between average temperature, total rainfall, and cholera incidence. We focused on districts located near CSD sentinel sites with reported cholera cases since 2022. Our analysis dataset included 2,298 records of cholera incidence, with an average of 32.8 cases between January 2022 and May 2024. Data were collected and organized on a weekly basis, covering the 52 epidemiological weeks (Epi weeks) for comprehensive analysis. The summarized data indicated that all study districts experienced cholera outbreaks in 2023 and 2024. Notably, Bahir Dar Zuria district reported the highest total number of cholera cases (587), followed by Dubti district (450). Of the total cholera cases reported, approximately 63.7% occurred between Epi week 29 and 42. In contrast, between Epi week 15 and 21, about 17.5% of the cases were reported. During the study period, observed mean temperature ranged from 10.4°C to 35.1°C, and total rainfall ranged from 0 mm to 584 mm. We identified two distinct cholera transmission seasons. The first and major one was the wet season (*Kiremt*), June–September, characterized by peak heavy rains. This monomodal cholera incidence pattern was observed in Bahir Dar Zuria, Dera (AM), Dessie, Dilla, and Hawassa districts. The second and bimodal season pattern was observed, encompassing the secondary wet season (*Belg*), February–May, with occasional showers, followed by the wet season (*Kiremt*). This pattern was observed in Dubti, Gode, Dire Dawa, Jinela, Kombolcha, Hakim, Semera Logia, and Wondo Genet districts (Figure 2).

**Figure 3:**
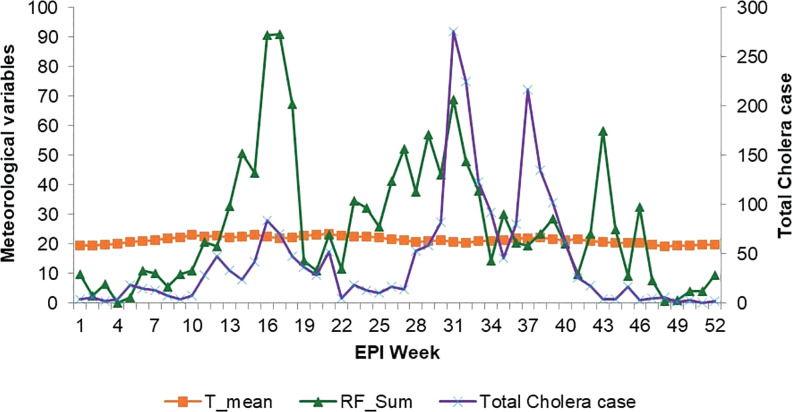
Total cholera distribution with rainfall and temperature in the study districts, Ethiopia.

Figure 3 illustrates the combined trend of cholera cases across all districts, alongside the average mean temperature and total rainfall. The distribution of temperature and rainfall correlated closely with the distribution of cholera cases. Cholera seasonality showed distinct patterns. Most areas generally had a higher excess risk of cholera occurrence in August–October, particularly the monomodal rainfall-benefiting areas (Figure 3).

**Figure 4:**
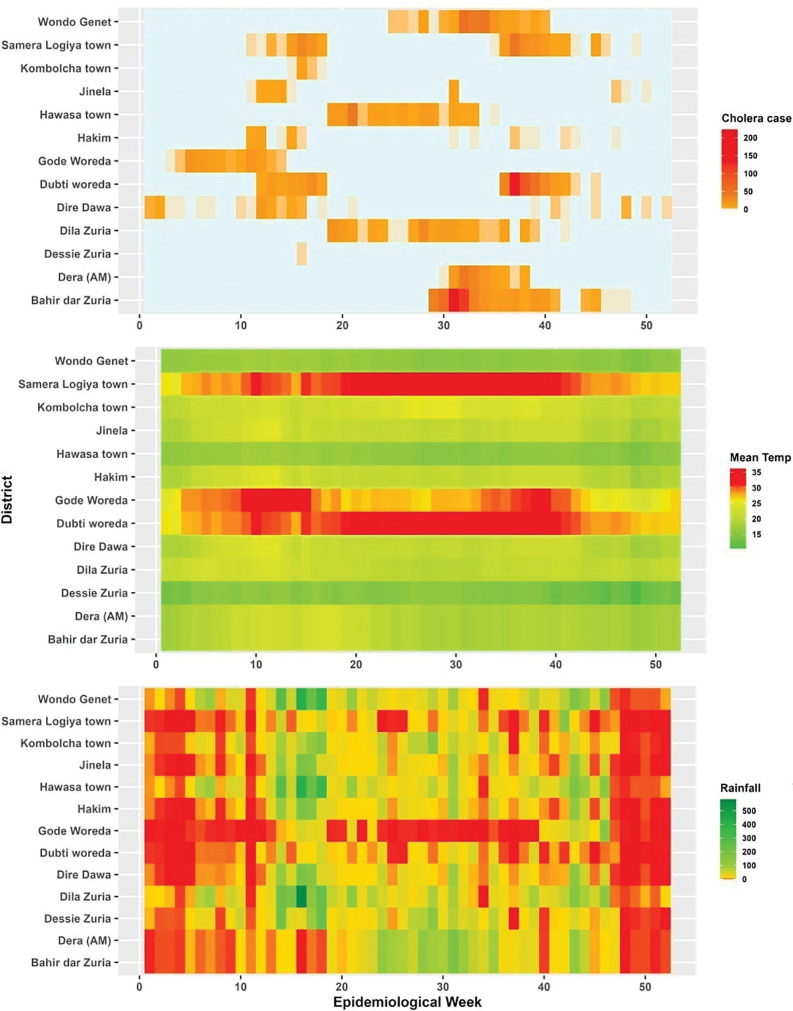
Heatmap of cholera incidence in relation to temperature and rainfall in the study districts, Ethiopia.

To better understand the association between cholera cases and climate data, Figure 4 presents a heatmap of all variables. Darker shades represent higher cholera cases, rainfall, or mean temperature, with each row corresponding to a district and each column to an epidemiological week. It shows that most districts experienced cholera outbreaks corresponding to the months April–September. This period coincides with the rainy season in most parts of the country. However, there is significant variation in temperature distribution between districts (Figure 4).

El Niño/ENSO could also impact climate-induced diseases. In the study area, cholera distribution was associated with ENSO (SST). Cholera cases increase during positive ENSO events, particularly when the ENSO index rises above 0.5°C (warm phase). Notably, cholera resurgence aligns with two peak seasons: July–September and February–May in several districts.

To supplement the descriptive analysis, we conducted a correlation analysis. Table 2 summarizes the correlation results at four different lag times and p-values. The four lag times, Lag 0, Lag 1, Lag 2, and Lag 3, represent the effects of rainfall or temperature observed at different intervals. In the same week (Lag 0, no delay), after one week (Lag 1), after two weeks (Lag 2), and after three weeks (Lag 3). The analysis indicates that the effects of rainfall and temperature on cholera incidence vary across districts. Table 2 indicates that of the 13 selected districts, 2/13 are only correlated with rainfall, and 8/13 (62%) are correlated with temperature. Additionally, 2/13 of the districts are correlated with both temperature and rainfall, while 1/13 is not correlated with any of the climate factors. The monomodal cholera transmission areas show a strong positive correlation with rainfall at various lag times. For example, Bahir Dar Zuria shows moderate positive correlation at Lag 0 [r = 0.618, p < 0.001], Dera AM has a 59% correlation at lag 1, and Dessie has a 56% correlation at lag 2, which demonstrate significant correlations with rainfall. Similarly, bimodal cholera transmission areas show positive correlations with temperature at different lag times. For instance, Dire Dawa has a 31% correlation at lag 3, Gode 35% at lag 0, Dubti 37% at lag 1, Hakim 48% at lag 0, and Semera Logia 34% at lag 1, all showing significant correlations with temperature. In contrast, Hawassa Zuria exhibits weak and inconsistent correlations with both rainfall (r = 0 across all lags, p > 0.3) and temperature (r ranging from −0.029 to −0.134, p > 0.3). This indicates that there is district-specific variability that may reflect unmeasured, confounding local factors such as WaSH coverage, vaccination status, and the like (Table 2).

**Table 2:** Correlation values between cholera incidence and rainfall and temperature at lag 0 to 3 weeks in selected districts, Ethiopia.

DISTRICT	CLIMATE VARIABLE	CORRELATION (P-VALUE)			
		LAG 0 (AT SAME WEEK)	LAG 1 (AFTER 1 WEEK)	LAG 2 (AFTER 2 WEEKS)	LAG 3 (AFTER 3 WEEKS)
**Bahir Dar Zuria**	Rainfall	***0.618(0.001)***	***0.519(0.001)***	***0.439(0.001)***	***0.468(0.001)***
	Temperature	***–0.264(0.025)***	–0.235(0.053)	–0.219(0.075)	–0.216(0.082)
**Dera (AM)**	Rainfall	***0.397(0.001)***	***0.594(0.001)***	***0.566(0.001)***	***0.551(0.001)***
	Temperature	***–0.239(0.048)***	***–0.267(0.028)***	***–0.249(0.042)***	–0.239(0.053)
**Dessie**	Rainfall	–0.008(0.95)	0.019(0.876)	***0.566(0.001)***	–0.019(0.881)
	Temperature	0.191(0.116)	0.074(0.55)	0.032(0.797)	0.174(0.163)
**Dilla**	Rainfall	0.01(0.933)	–0.008(0.946)	0.022(0.86)	0.075(0.552)
	Temperature	***–0.384(0.001)***	***–0.39(0.001)***	***–0.333(0.006)***	***–0.315(0.01)***
**Dire Dawa**	Rainfall	–0.051(0.677)	–0.103(0.402)	–0.049(0.694)	–0.166(0.182)
	Temperature	0.225(0.064)	***0.303(0.012)***	***0.306(0.012)***	***0.316(0.01)***
**Dubti**	Rainfall	–0.017(0.887)	–0.017(0.893)	0.091(0.464)	0.116(0.355)
	Temperature	***0.356(0.003)***	***0.372(0.002)***	***0.35(0.004)***	***0.326(0.007)***
**Gode**	Rainfall	–0.158(0.195)	–0.158(0.197)	–0.155(0.209)	–0.151(0.227)
	Temperature	***0.352(0.003)***	***0.307(0.011)***	0.236(0.054)	0.083(0.506)
**Hakim**	Rainfall	0.015(0.905)	0.01(0.934)	–0.018(0.883)	–0.135(0.279)
	Temperature	***0.486(0.001)***	***0.444(0.001)***	***0.422(0.001)***	***0.473(0.001)***
**Hawassa Zuria**	Rainfall	0(0.836)	0(0.757)	0(0.865)	0(0.301)
	Temperature	–0.092(0.836)	–0.134(0.757)	–0.029(0.865)	–0.049(0.301)
**Jinela**	Rainfall	0.178(0.143)	–0.077(0.533)	–0.129(0.299)	–0.091(0.469)
	Temperature	0.108(0.375)	***0.302(0.012)***	***0.344(0.004)***	***0.325(0.008)***
**Kombolcha**	Rainfall	***0.259(0.032)***	0.136(0.268)	0.231(0.06)	0.082(0.513)
	Temperature	0.017(0.89)	–0.004(0.975)	–0.07(0.575)	–0.003(0.98)
**Semera Logia**	Rainfall	0.04(0.744)	–0.004(0.975)	0.094(0.448)	0.149(0.232)
	Temperature	***0.321(0.007)***	***0.342(0.004)***	***0.302(0.013)***	***0.303(0.014)***
**Wondo Genet**	Rainfall	–0.134(0.273)	–0.053(0.668)	–0.047(0.704)	0.047(0.707)
	Temperature	***–0.258(0.032)***	***–0.291(0.016)***	***–0.299(0.014)***	***–0.312(0.011)***

*Note: values in Bold and Italic indicate significant value at P-value of 0.05.*

### Observed thresholds associated with cholera peaks

To understand how temperature and rainfall conditions might lead to the rapid growth and transmission of *V. cholerae* and the onset of epidemic, we calculated thresholds. These thresholds represented the 33-year historical mean temperature and rainfall plus or minus anomalies observed during cholera peaks. Table 3 summarizes observed thresholds of temperature and rainfall for outbreak risk and the proliferation of *V. cholerae* at the four lag times. These values can serve as early warning indicators for outbreaks in the corresponding districts. Values in italics indicate significant correlation (Table 3).

**Table 3:** Observed associations of temperature and rainfall parameters for *Vibrio Cholerae* proliferation at epidemic onset in selected districts, Ethiopia.

*DISTRICT*	CLIMATE VARIABLE	THRESHOLDS FOR PROLIFERATION OF *VIBRIO CHOLERAE*			
		LAG 0 (AT SAME WEEK)	LAG 1 (AFTER 1 WEEK)	LAG 2 (AFTER 2 WEEKS)	LAG 3 (AFTER 3 WEEKS)
**Bahir-Dar Zuria**	Rainfall	***106.2–15.3***	***90.1+31.3***	***85.3–30.1***	***80.8–1.3***
	Temperature	***16.9 +1.5***	17.1+1.1	17.6+0.6	18.3+0.8
**Dera (AM)**	Rainfall	***112.0–26.4***	***106.2–15.3***	***90.1+31.3***	***85.3–30.1***
	Temperature	***16.8+1.7***	***16.9+1.5***	***17.1+1.1***	17.6+0.6
**Dessie**	Rainfall	14.0+16.7	13.8+22.9	***19.2+188.1***	11.8+8.8
	Temperature	15.3+1.1	15.4–0.3	15.3–0.8	15.3+0.7
**Dilla**	Rainfall	56.5–40.4	43.9+4	26.7+52.3	24.8+63.5
	Temperature	***22.5–0.3***	***23.0–1.7***	***23.7–2.5***	***23.9–2.8***
**Dire Dawa**	Rainfall	11.9+1.5	21.9+9.3	36.1–31.4	34.8–21.5
	Temperature	21.0+1.1	***20.9+1.4***	***20.7+1.6***	***20.6+1.2***
**Dubti**	Rainfall	18.7–17.6	21.6+6.6	21.7–18.9	29.6+15.1
	Temperature	***31.4+2.7***	***31.2+1.7***	***31.1+2.1***	***30.8+1.3***
**Gode**	Rainfall	0.3–0.3	0.3–0.2	0.3–0.3	NA
	Temperature	***27.5+1.1***	***27.0–1.2***	26.6–1.9	NA
**Hakim**	Rainfall	32.8+66.2	32.2–25.5	30.2–19.2	30.6–10.1
	Temperature	***20.4–0.2***	***20.4+0.8***	***20.7+0.1***	***20.9–0.1***
**Hawassa Zuria**	Rainfall	57.2+94.9	53.1–22.1	50.9–36.7	40.7–1.9
	Temperature	16.3–0.6	16.7+0.4	16.9–0.4	17.2–0.9
**Jinela**	Rainfall	3.9–3.9	4.7–4.2	4.4+45.6	6.4–5.2
	Temperature	19.0+0.3	***19.2+0.7***	***19.4–0.3***	***19.6–0.4***
**Kombolcha**	Rainfall	***15.7+22.9***	21.3+65.9	16.8+13.0	18.8–4.4
	Temperature	23.2–1.1	23.0–2.0	22.7–0.4	22.5+0.8
**Semera Logia**	Rainfall	21.6+6.6	21.7–18.9	29.6+15.1	29.1–15.8
	Temperature	***31.2+1.7***	***31.1+2.1***	***30.8+1.3***	***30.6+2.5***
**Wondo Genet**	Rainfall	31.3–16.8	29.8+3.5	27.8+31.9	33.7–6.4
	Temperature	***15.0+1.0***	***15.3+0.6***	***15.7+0.4***	***15.9–0.1***

*Note:* Threshold = 33 year mean +/- Anomaly.

## Discussion

This case study explores the relationship between climate variables and cholera incidence to identify potential indicators for an EWS. The findings highlight a significant concentration of cholera cases within specific epidemiological weeks, indicating a potential seasonal pattern influenced by climatic conditions. Understanding these associations can aid in forecasting and implementing timely preventive measures, particularly during high-risk periods. The findings also demonstrate the importance of accounting for district-specific weather variations in predicting cholera risk. Seasonal transmission patterns revealed two distinct cholera transmission seasons. Some districts primarily experienced peaks in cholera cases during the heavy rainfall from June to September (monomodal), while others experienced a secondary peak during February–May (bimodal). The observed variation across districts with monomodal patterns, like Bahir Dar Zuria, may be more influenced by heavy rainfall due to reliance on surface water sources, while bimodal districts could reflect ENSO-driven anomalies. Due to the short incubation period of *V. cholerae* and a relatively brief lag of less than a month following temperature and precipitation changes, delays in surveillance can significantly hinder effective outbreak responses [16]. Thus, consideration of lag times is crucial as it could inform improvements in early warning [11].

Temperature appears to have a varied impact across districts. In most areas, it was positively correlated and had a moderate positive effect on cholera cases. The impacts occurred immediately or after a delay, persisting for a few weeks. This finding aligns with a study reporting a three-week lag [16] and a systematic review reporting that the impact of temperature on cholera was lagged by two weeks in Cameroon, three weeks in Peru, and four weeks in Bangladesh and Tanzania [11]. The observed difference in lag times indicates a delayed response to temperature changes in driving outbreaks. This delay may be due to factors such as the incubation period, as well as other indirect effects like WaSH coverage and hygiene behavior. Conversely, some regions showed a weak negative correlation between temperature and cholera cases, potentially indicating the strong influence of other variables beyond weather variations. Rainfall affects cholera outbreaks both immediately and with delays. The strongest correlations typically occurred in the same week, with lingering effects observed in subsequent weeks. This aligns with a systematic review reporting rainfall lag times of 0–4 months in India and Bangladesh, 6 days in Haiti, and 15 to 30 days in Cameroon and Peru [11]. These delays may result from factors like incubation period, water contamination, or limited access to clean water following heavy rain [10, 11, 29]. In some areas, however, rainfall had a weak and inconsistent impact. While a slight correlation was observed in the same week, the effect on cases in later weeks was minimal and statistically insignificant.

With regard to ENSO, the alignment of cholera resurgence with warm phases (SST > 0.5°C) in some districts suggests a potential synergistic effect between ENSO-induced temperature anomalies and local rainfall patterns. Previous studies have linked ENSO events to increased incidence through altered precipitation and flooding [11]. These findings show the importance of integrating ENSO monitoring into cholera surveillance and response strategies. Overall, the reported cholera-weather associations significantly vary due to underlying variation across districts with respect to socio-economic, health infrastructure, behavioral, WaSH, population density, and other contextual and modifying factors. However, the findings may still be relevant for understanding localized patterns of cholera transmission and informing EWS.

Quantifying changes to cholera cases attributable to forecasting is challenging and requires further study. Nonetheless, preliminary reports indicate forecasting can potentially inform response by detecting outbreaks early and identifying areas at risk, leading to timely deployment of rapid response teams for investigations and targeted interventions, including case identification, isolation, and treatment. It can also enhance risk communication and community engagement through targeted campaigns and specific WaSH interventions [30].

The effects of climate conditions on health are strongly mediated by socio-economic and environmental vulnerabilities, creating a complex pathway [3]. Projections for Ethiopia indicate an increase in population at risk of riverine flooding due to extreme weather events [31]. Unpredictable weather patterns also increased migration and displacement, a known cholera outbreak risk factor, with cholera hotspots facing a dual burden from internally displaced persons and refugees [31]. Erratic rains have also been linked to persistent food insecurity and malnutrition [7, 32, 33]. To mitigate the impacts of climate change on droughts and flooding, the Ethiopian Disaster Risk Management Commission issues district-level alerts on flooding and drought along with vulnerability and hotspot maps based on climate data sourced from the EMI. The commission incorporates floods and droughts in its national multi-hazard plans, mainly for early response efforts, in cooperation with international agencies. However, flooding prediction is data-limited and has yet to be harmonized with cholera data, which is essential to link flooding risk and cholera incidence in specific geographic locations.

Several institutional drivers, particularly surveillance and EWS factors, impact cholera. In alignment with the global cholera roadmap 2023 [34], improving cholera surveillance is a core component of the NCP as one of the six pillars critical to achieving the elimination of 90% of cholera by 2028 [7, 35]. Data from routine surveillance provide essential information for forecasting and alerting efforts. However, the cholera surveillance system faces challenges, including poor data quality, underreporting, weak digitization, insufficient personnel, and infrastructure damage from political instability [7, 33, 35]. As a result of these constraints, the status of data utilization for action and decision-making is relatively low. The cholera early warning and forecasting system also suffers from similar challenges: 1) Data interoperability: Integrating data from various sources is difficult due to incompatible formats. 2) Manual data integration: Relying on manual processes delays early warnings. 3) Lack of key variables: Incomplete socio-economic and WaSH data undermines forecasts. 4) Financial constraints: Insufficient funding hampers system upgrades. 5) Skill shortages: Lack of trained professionals for data analysis and forecasting.

In order to test, deploy, and evaluate cholera intervention measures, it is crucial to compile comprehensive data on climate variability trends, risk factors, and distribution of cases. Hence, it is vital to examine surveillance data sources and tools that can be integrated to enhance data analysis [27]. In Ethiopia, epidemiological cholera data are routinely compiled from various sources, with the Health Management Information System (HMIS) and District Health Information System 2 (DHIS 2) being primary sources [7]. Districts currently use these platforms for daily and weekly cholera reports rather than forecasting. However, practical usage challenges persist, including poor infrastructure, inconsistent data quality, and suboptimal staff skills [36]. The rollout of the DHIS2 system has been slow and lacks integrated meteorological data necessary for effective forecasting. Although not yet implemented locally, the DHIS2 climate app has the potential to utilize the ERA5-Land climate dataset to provide daily district-level weather data, addressing issues of suboptimal local climate data reporting and completeness.

A centralized database is necessary for managing collected data from various sources. Implementing algorithms to analyze spatiotemporal patterns of cases and environmental parameters may be crucial for detecting early cholera outbreak signs. An interoperable system with satellite and GPS integration can ensure accurate geo-location of data points for weather parameters, drought and flood-affected areas, and cholera cases. Using standardized data formats and protocols can guarantee compatibility between stakeholders like the EMI, Disaster Risk Commission, Ethiopia Public Health Institute, and NGOs. While the overall availability of climate data suitable for national and local analyses has been improving [21], greater emphasis should be placed on enhancing data granularity and accessibility.

Studies have demonstrated the effectiveness of both simple time series and artificial neural networking for forecasting [16, 37, 38], as well as mechanistic methods such as the Early Warning, Alert and Response System (EWARS) for predicting CSD [39]. The EWARS has shown good sensitivity and predictive value across different study settings, particularly CSD, such as dengue fever [39]. In Ethiopia, data from sentinel sites have validated the EWARS modeling and forecasting tool for malaria and meningitis [4]. The EPHI has begun exploring EWARS for forecasting cholera outbreaks alongside climate and environmental data [4]. However, predictive modeling faces challenges due to limited data collection, integration, computing, and expertise.

### Lessons learned

This study demonstrates the potential of climate data to enhance cholera forecasting. It also reveals limitations due to incomplete datasets and sectoral hurdles. Simple correlation-based analysis can potentially be a foundational approach for EWS in high-risk, resource-limited settings like Ethiopia, where it can identify climate triggers despite weak healthcare infrastructure. High-quality data and cross-sector collaboration are required for effective forecasting, demonstrating the need to strengthen existing systems and training the health workforce while improving infrastructure. Other similarly affected regions can use these insights to inform their forecasting strategies tailored to their specific health system contexts.

### Limitations

The study has several limitations. The use of correlation alone assumes linearity, while cholera has complex transmission dynamics that climate variables alone do not fully capture. Key district-specific socio-economic and WaSH indicators were excluded due to irregular collection, reporting, and missing data. This potentially overestimates the direct role of climate variables. Such unaccounted confounding factors may have skewed reported associations. The three-week lag analysis may not fully capture delayed climate effects. Advanced predictive analysis with model validation was not feasible due to the lack of complete long-term cholera data and the misclassification of cholera cases as acute watery diarrhea (AWD).

## Conclusion and recommendation

The findings demonstrate the potential of harnessing climate data, particularly in cholera-endemic regions prone to drought and flooding, to inform EWS. The reported rainfall and temperature thresholds, though preliminary, could serve as initial triggers for alerts, with refinement through real-time data. Further development and validation are needed to translate these insights into operational tools. Collaboration among stakeholders for open data sharing is essential for adaptation research aimed at forecasting and early warning of outbreaks. It is essential to utilize diverse data sources and expertise from multi-disciplinary professionals, such as biostatisticians, epidemiologists, and bio-meteorology experts. Key challenges include consolidating multiple data sources and addressing data access hurdles. Moving toward a multi-sectoral, interoperable system is recommended, focusing on consolidating data exchange and forecasting platforms such as DHIS2 and EWARS, along with climate data.

## Data Availability

All datasets used to support the conclusions of this paper are available from the corresponding author on request.
